# Preparation and Property Analysis of Antibacterial Fiber Membranes Based on Hyperbranched Polymer Quaternary Ammonium Salts

**DOI:** 10.3390/ma17153761

**Published:** 2024-07-30

**Authors:** Jiehui Zhu, Ying Guo, Lirong Yao, Gangwei Pan, Desuo Zhang, Jianwei Yang

**Affiliations:** 1 College of Textile and Clothing, Nantong University, Nantong 226019, China; 17317506847@163.com (J.Z.); 16651322293@163.com (J.Y.); 2 National & Local Joint Engineering Research Center of Technical Fiber Composites for Safety and Protection, Nantong University, Nantong 226019, China; pangangwei@ntu.edu.cn; 3 College of Textile and Clothing Engineering, Soochow University, Suzhou 215123, China; dszhang@suda.edu.cn

**Keywords:** hyperbranched polymer, quaternary ammonium salts, fiber membranes, antibacterial property

## Abstract

Due to their excellent properties, antimicrobial fiber membranes are widely applied in bioprotective materials. This work addresses the preparation of thermoplastic polyurethane (TPU)-based fiber membranes with active antimicrobial properties. 2-hydroxypropyl trimethyl ammonium chloride-terminated hyperbranched polymer (HBP-HTC) was synthesized and used as an antimicrobial agent. The fiber membranes were obtained by electrospinning a mixed solution of HBP-HTC and TPU. Different electrospinning conditions were investigated, such as the spinning voltage and drum rotation speed. The fiber membrane prepared under a 22 kV anode voltage and 100 rpm rotation speed had an average fiber diameter of 1.66 μm with a concentrated diameter distribution. Antibacterial tests showed that when the fiber membrane was loaded with 1500 mg/kg of HBP-HTC, the antibacterial rates of *E. coli* as well as *S. aureus* both reached 99.99%, exhibiting excellent proactive antimicrobial performance. Moreover, the protective performance of the fiber membrane was outstanding, with a filtration efficiency of 99.9%, a hydrostatic pressure resistance greater than 16,758 Pa, and a moisture permeability of 2711.0 g⋅(m^2^⋅d)^−1^.

## 1. Introduction

In recent years, there has been an increase in the demand for antimicrobial materials, driven by improvements in living standards and the growing impact of biosecurity threats [1]. Quaternary ammonium salts, known for their physical sterilization, low cost, broad-spectrum antimicrobial effects [2], and extensive applications, are extensively used in a variety of fields such as crop protection, disinfection in public places, and the sterilization of medical devices. The antibacterial mechanism is generally considered to involve the adsorption of positively charged cations onto the negatively charged surface of bacteria, followed by the penetration of the quaternary ammonium salt side chains into the cell membrane, leading to the leakage of intracellular substances and, consequently, bacterial inactivation [3]. The classification of quaternary ammonium salts encompasses mono-, di-, tri-, and poly-quaternary ammonium salts, as well as hyperbranched polymer quaternary ammonium salts. Hyperbranched polymer quaternary ammonium salts, distinguished by their straightforward synthesis and beneficial attributes such as enhanced solubility and pronounced reactivity, have garnered extensive application across a spectrum of domains, including water treatment [4,5,6], filtration media [7,8,9], and the sterilization and preservation of foodstuffs [10].

Presently, the conventional approach to fabricating antibacterial fiber membranes entails the incorporation of an antibacterial agent into the spinning solution, followed by direct spinning [11]. For instance, in the study [12] by Karagoz et al., ZnO nanorods and silver nanoparticles were homogeneously dispersed within a methyl methacrylate (PMMA) matrix, yielding PMMA/ZnO–Ag NFs fiber membranes via the electrospinning technique. This compound demonstrates potent bactericidal activity against various bacteria, including Gram-negative and Gram-positive strains. Also, it exerts inhibitory effects on coronaviruses and influenza viruses, underscoring its significant promise for applications within the protection domain. Chen et al. [13] initially synthesized copper nanoparticles via the microwave-assisted liquid-phase reduction method, subsequently integrating them with a poly(ε-caprolactone)/gelatin blend to yield Cu NPs/PCL/Gt fiber membranes through the application of electrospinning technology. Subsequent antibacterial assays demonstrated that these composite fiber membranes exhibited superior antibacterial efficacy. In a recent study [14], Xia et al. synthesized silver nanoparticles, approximately 10 nm in diameter, employing a chemical in situ reduction technique. Subsequently, these nanoparticles were homogeneously dispersed within a polyurethane matrix, yielding silver-embedded nanofiber membranes via the electrospinning process. The resultant membranes exhibited exceptional antibacterial efficacy, with antibacterial rates of 99.99% against both Gram-negative bacillus and Gram-positive bacteria. The evidence suggests a significant enhancement in the protective performance of the material when antimicrobial substances are incorporated into the spinning solution matrix.

The use of nanoparticles as antimicrobial additives for the fabrication of bioprotective materials has been extensively reported. Achieving the uniform dispersion of nanoparticles in solutions has remained a significant challenge. Hyperbranched polymer quaternary ammonium salts possess excellent solubility, leading to good dispersion when added to a spinning solution. The resulting fiber membranes exhibit minimal variation in antimicrobial performance. To date, the application of hyperbranched polymer quaternary ammonium salts in bioprotective materials has been scarcely documented. Zhao et al. [7] incorporated hyperbranched polymer quaternary ammonium salts into polyvinylidene fluoride (PVDF) to prepare dendritic nanofiber filter materials. The findings demonstrated that the hyperbranched polymer quaternary ammonium salts promoted the generation of dendritically ultra-fine nanofibers, thereby endowing the fiber membrane with enhanced air filtration capabilities and imparting it with exemplary antimicrobial properties. Chen et al. [15] chemically grafted quaternary ammonium salt (QAS) molecules onto biodegradable polylactic acid (PLA) substrates, resulting in PLA-QAS membranes with excellent broad-spectrum bactericidal properties, achieving an antibacterial rate greater than 99.99%. These membranes maintain their outstanding bactericidal performance and excellent durability, even at high water filtration rates. It can be inferred that hyperbranched polymer quaternary ammonium salts, due to their superior electrical conductivity and antimicrobial properties, can enhance the value of fiber membranes, offering new avenues for the preparation of high-performance bioprotective materials and water filtration media.

In this paper, we innovatively used HBP-HTC as an antibacterial agent, which solved the problem of the poor dispersion of traditional nano-particle antibacterial agents in the polymer matrix. HBP-HTC was added to a thermoplastic polyurethane (TPU) spinning solution as an antibacterial agent. Subsequently, the fabrication of the HBP-HTC/TPU fiber membrane was conducted through the use of the electrospinning technique. After the incorporation of HBP-HTC, there was no significant alteration to the microstructure of the TPU fibers. By adjusting the electrospinning process parameters, a concentrated distribution of fiber diameters within the fiber membrane was achieved. The fiber membrane was subjected to antibacterial performance testing, demonstrating excellent bacteriostatic effects. Additionally, the results from the filtration efficiency, hydrostatic pressure resistance, and moisture permeability indicate that the fiber membrane possesses superior bioprotective properties, holding promise for the development of novel medical protective composite materials.

## 2. Materials and Methods

### 2.1. Materials

The materials utilized included thermoplastic polyurethane elastomer (TPU, Shanghai Yuanneng Plastics Co., Ltd., Shanghai, China); 2-hydroxypropyl trimethyl ammonium chloride-terminated hyperbranched polymer (HBP-HTC, synthesized in the laboratory); deionized water (prepared in the laboratory); N, N-dimethylformamide (DMF); acetone (analytical grade, Zhejiang Petrochemical Co., Ltd., Zhoushan, China); Escherichia coli; Staphylococcus aureus (Shanghai Luwei Technology Co., Ltd., Shanghai, China); Nutrient Broth; Nutrient Agar (NA); and Phosphate-Buffered Saline (PBS) (Hangzhou Baisi Biotechnology Co., Ltd., Hangzhou, China).

### 2.2. Synthesis and Characterization of HBP-HTC

**Figure 1:**
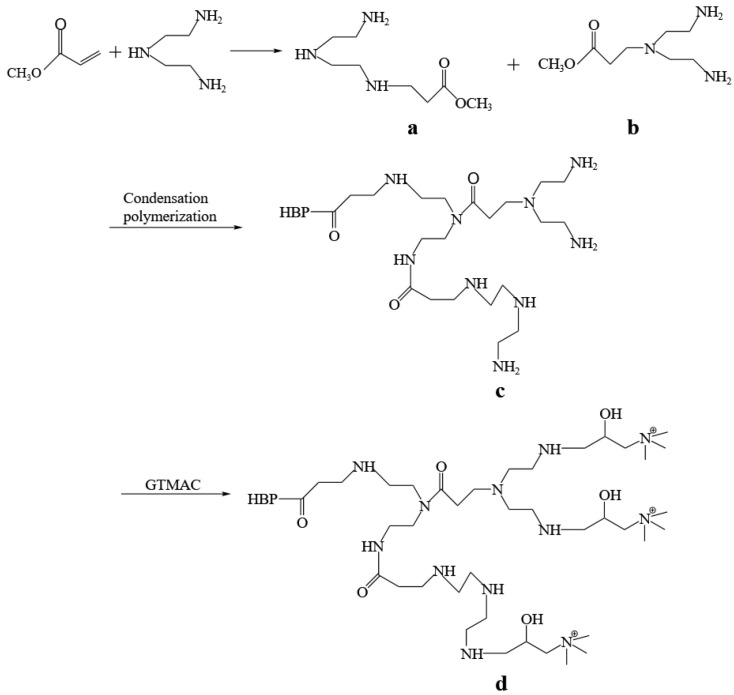
Synthetic roadmap of HBP-HTC ((**a**) AB_3_; (**b**) AB_2_; (**c**) HBP-NH_2_; (**d**) HBP-HTC).

HBP-HTC was prepared by the methodology delineated in the literature [16], as shown in Figure 1. The brief preparation process is as follows: Under a nitrogen atmosphere, a mixed solution of methyl acrylate and methanol was added dropwise to diethylenetriamine. After the reaction, a pale-yellow, transparent AB_3_ and AB_2_ monomer was obtained (Figure 1a,b). After the removal of the methanol, the temperature was raised to 150 °C, and the reaction continued under reduced pressure to yield a viscous, pale-yellow, amine-terminated hyperbranched polymer, HBP-NH_2_ (Figure 1c). This compound was dissolved in water, and an aqueous solution of 2,3-epoxypropyltrimethylammonium chloride was added dropwise. Once the reaction was complete, the product was separated and purified to yield a pale-yellow, solid HBP-HTC (Figure 1d).

Fourier Transform Infrared Spectroscopy analysis was performed on the intermediate monomers of the AB_3_ and AB_2_ types, as well as on the HBP-NH_2_ and HBP-HTC samples, using an infrared spectrometer (FTIR; Thermo Nicolet iS50, Thermo Fisher Scientiffc, Waltham, MA, USA). Nuclear magnetic resonance measurements, including ^1^H-NMR and ^13^C-NMR, were conducted on the HBP-NH_2_ and HBP-HTC samples using an NMR spectrometer (NMR; Unity Ivova 300M, Ivova, Fairfax, VA, USA) [17,18].

### 2.3. The Preparation and Characterization of the Spinning Solution

The TPU spinning solution, obtained by mixing TPU with a 1:1 mass mix solution of DMF and acetone, was stirred magnetically at 85 °C until the TPU dissolved [19]. The mass fraction of TPU in the solution was 27% [20]. Subsequently, varying amounts of HBP-HTC were incorporated into the TPU spinning solution to achieve mass concentrations of 0, 1000, 1500, and 2000 mg/kg for the HBP-HTC/TPU mixture. Maintained at 85 °C in heated water and subjected to constant mechanical stirring, the solutions were subjected to an additional three-hour heating period.

The homogeneous spinning solution was characterized by following specific standards. Following the GB/T 10247-2008 “Viscosity Test Method”, the viscosity of the spinning solvent was determined by means of a rotational rheometer (MCR102, Anton Paar GmbH, Graz, Austria). By referring to the GB/T 11007-2008 “Conductivity Meter Test Method”, the electrical conductance of the spinning solution was evaluated utilizing a conductivity meter (Five-Easy, Hongrun Precision Instruments Co., Ltd., Nanping, China). By adhering to the GB/T 22237-2008 “Determination of Surface Tension by Surface Tension Meter for Surfactants”, the surface tensions of the spinning solvent solutions were measured using a surface tension meter (DCAT11, Deca Precision Instruments Co., Ltd., Shenzhen, China).

### 2.4. Preparation and Characterization of HBP-HTC/TPU Fiber Membranes

A 5 mL syringe was employed, loaded with the HBP-HTC/TPU spinning solvent, and fitted with a flat-tip needle with an inside dimension of 0.41 mm [21]. Four types of TPU fiber membranes with varying HBP-HTC contents (0, 1000, 1500, and 2000 mg/kg) were produced via electrospinning. Following the spinning process, the fiber membranes were transferred to an oven set at 60 °C for an hour to dry them and remove residual solvents. The electrospinning process parameters were set as follows: the injection dosage was 3 mL, the pulling rate was 1 mL/h, the drum rotation speed was 100 ± 5 rpm, the voltage was 22 ± 0.5 kV, the receiving distance was 20 ± 2 cm, the ambient temperature was 18 ± 5 °C, and the humidity was 40 ± 10% [22,23,24].

The morphological observations of the fiber membranes with HBP-HTC contents of 0, 1000, 1500, and 2000 mg/kg were conducted using a scanning electron microscope (SEM; ZEISS Gemini SEM300, Carl Zeiss Company, Oberkochen, Germany). The fiber diameters were subjected to statistical analysis using Nano Measurer 1.2 software, resulting in the generation of diameter distribution graphs. An energy-dispersive spectrometer (EDS; ZEISS Gemini SEM300, Oberkochen, Germany) was employed to conduct an elemental analysis of the fiber membrane with an HBP-HTC content of 1500 mg/kg. X-ray photoelectron spectroscopy (XPS; Thermo Scientiffc K-Alpha+, Waltham, MA, USA) was employed for the elemental analysis of the fiber membranes with HBP-HTC contents of 0 and 1500 mg/kg.

### 2.5. The Optimization of the Preparation Process of HBP-HTC/TPU Fiber Membranes

1. The Effect of the Spinning Voltage on the Surface Structure of the HBP-HTC/TPU Fiber Membrane

The content of HBP-HTC in the TPU spinning solution was controlled at 1500 mg/kg, with the drum rotation speed set to 100 rpm. The spinning voltage was adjusted to 18, 20, 22, and 24 kV to prepare the fiber membranes via electrospinning, resulting in HBP-HTC/TPU fiber membranes fabricated under different spinning voltages. The surface morphologies of the fibers were observed via SEM at various spinning voltages, and diameter distribution statistics were performed in order to identify the optimal spinning voltage.

2. The Effect of the Drum Rotation Speed on the Surface Structure of the HBP-HTC/TPU Fiber Membrane

Under the optimal spinning voltage mentioned above, with the HBP-HTC content maintained at 1500 mg/kg, the drum rotation speed was adjusted to 100, 500, and 1000 rpm to produce HBP-HTC/TPU fiber membranes at different drum rotation speeds. The surface morphology characteristics of the fibers at various drum rotation speeds were examined using SEM, and diameter distribution statistics were performed to identify the optimal drum rotation speed.

### 2.6. Antibacterial Tests

The antibacterial performance of the fiber membranes with HBP-HTC contents of 0, 1000, 1500, and 2000 mg/kg was evaluated in accordance with the revised GB/T 20944.3-2008 “Textiles—Evaluation of Antimicrobial Activity—Part 3: Oscillation Method [14]”, with the fiber membrane at 0 mg/kg serving as the blank control [25].

### 2.7. Protective Performance Tests

Tests for the hydrostatic pressure resistance, filtration efficiency, and moisture vapor transmission rate of the fiber membranes with HBP-HTC contents of 0 and 1500 mg/kg were conducted in accordance with the relevant standards, with the standard numbers being GB/T 4744—2013, GB 2626—2019, and GB/T 1037—2021 [26].

## 3. Results and Discussion

### 3.1. Preparation and Characterization of HBP-HTC

To characterize the synthesis process and molecular structure of HBP-NH_2_ and HBP-HTC, analyses were conducted using FTIR, ^1^H NMR, and ^13^C NMR.

**Figure 2:**
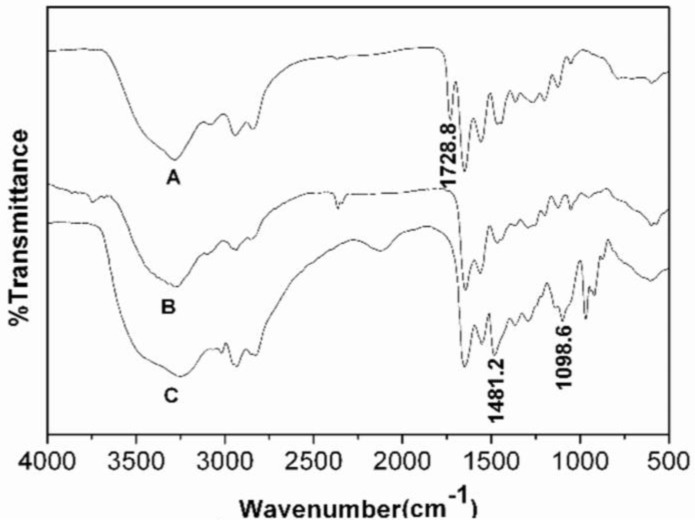
FTIR spectra (A. AB_2_ and AB_3_ type monomer; B. HBP-NH_2_; C. HBP-HTC).

Figure 2 presents the FTIR spectra of the AB_3_ and AB_2_ monomers (1 and 2), HBP-NH_2_, and HBP-HTC. The IR spectra of the three compounds exhibit a high degree of similarity, with a notable distinction being the appearance of a distinctive absorption peak at 1728.8 cm^−1^ for the carbonyl (C=O) group in the IR spectra of the AB_3_ and AB_2_ monomers [27], which is absent in the FTIR spectrum of HBP-NH_2_. This suggests that the ester bonds in the AB_2_ monomer fully reacted with the amine or imine groups to form amide bonds, confirming the synthesis of HBP-NH_2_. In the FTIR spectrum of HBP-HTC, peaks at 1481.2 cm^−1^ and 1098.6 cm^−1^ correspond to the characteristic absorption bands of δ_C-H_ for the -CH_3_ group and ν_C-OH_ for the secondary hydroxyl group in the quaternary ammonium salt side chain, respectively [16,28].

**Figure 3:**
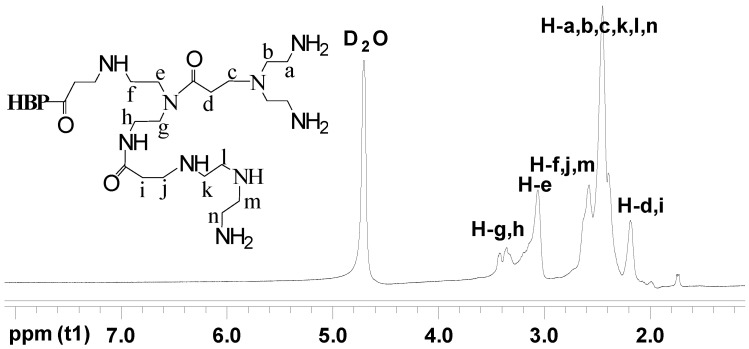
^1^H NMR spectrum of HBP-NH_2_.

**Figure 4:**
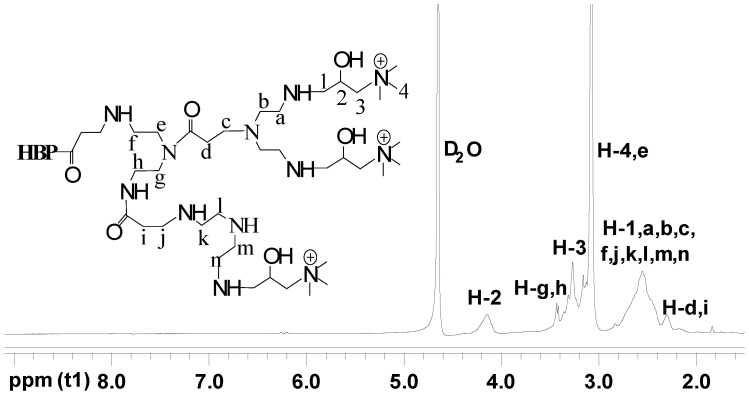
^1^H NMR spectrum of HBP-HTC.

Figure 3 and Figure 4 display the ^1^H NMR spectra of HBP-NH_2_ and HBP-HTC, respectively. There are significant differences between the two spectra. In the ^1^H NMR spectrum of HBP-HTC, a robust absorption peak attributed to the hydrogens of the three methyl groups in the quaternary ammonium salt side chain is discernible at δ = 3.1102. Additionally, an absorption peak for the hydrogen at the C_2_ position of the quaternary ammonium salt side chain emerges at δ = 4.1761.

**Figure 5:**
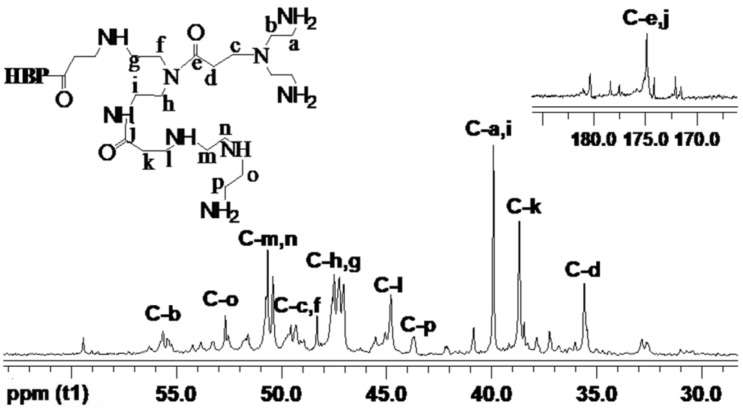
^13^C NMR spectrum of HBP-NH_2_.

**Figure 6:**
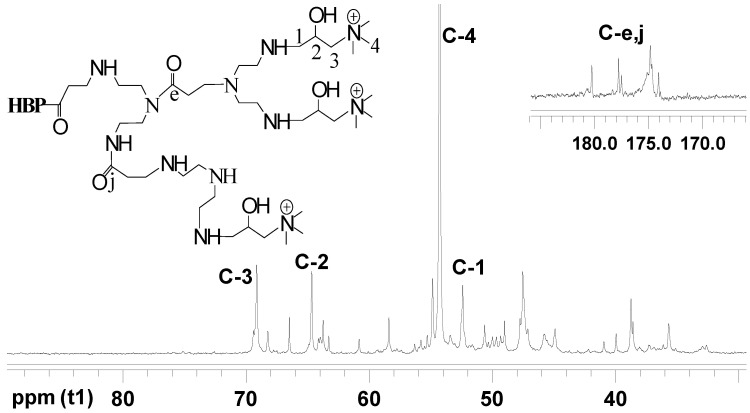
^13^C NMR spectrum of HBP-HTC.

Figure 5 and Figure 6 illustrate the ^13^C NMR spectra of HBP-NH_2_ and HBP-HTC, respectively. Although the grafting reaction complicates the ^13^C NMR spectrum of HBP-HTC, it is clear that the peaks at δ = 52.414, 64.007, and 69.397 ppm correspond to the C_1_, C_2_, and C_3_ carbons of the quaternary ammonium salt side chain, respectively. The peak at δ = 54.286 ppm corresponds to the three methyl carbons C_4_ attached to the nitrogen of the quaternary ammonium salt [29]. Based on the analysis of the aforementioned infrared spectroscopy and nuclear magnetic resonance spectroscopy, it can be inferred that a hydroxypropyl trimethylammonium chloride side chain was introduced onto the nitrogen in HBP-NH_2_, yielding the quaternary ammonium salt HBP-HTC.

### 3.2. The Preparation and Characterization of the Spinning Solution

As bioprotective membrane materials, the diameter and distribution of membrane fibers can significantly impact protective performance. In the electrospinning process, in addition to the spinning process parameters, the intrinsic properties of the spinning solution, such as electrical conductivity, surface tension, and viscosity, are also crucial influencing factors. Therefore, the stability of the spinning solution’s performance is an essential prerequisite for preparing fibers with a good morphology and excellent properties. This study assesses the impact of HBP-HTC addition on the smooth progression of the electrospinning process by testing the electrical conductivity, surface tension, and viscosity of the spinning solution.

**Figure 7:**
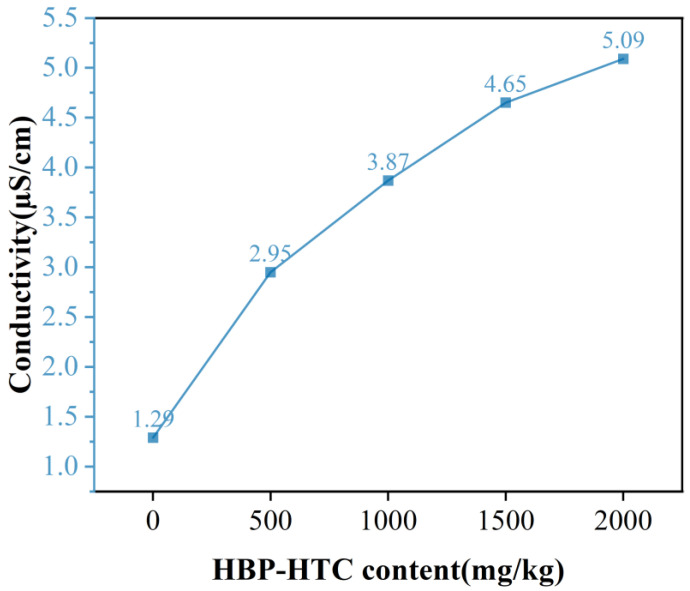
Conductivity of spinning solutions at different HBP-HTC content levels.

Figure 7 illustrates that as the content of HBP-HTC increases, the electrical conductivity of the spinning solution gradually increases, but the rate of increase diminishes. When the HBP-HTC content reaches 2000 mg/kg, the conductivity is 5.09 µS/cm. It is known from the literature [7] that fibers do not exhibit branching phenomena when the spinning solution’s electrical conductivity is around 6 µS/cm. In this experiment, the highest conductivity of the spinning solution was only 5.09 µS/cm. It can be seen that within the current range of the HBP-HTC content, its impact on the performance of the spinning solution is not significant, which is conducive to enhancing the stability of the electrospinning process.

**Figure 8:**
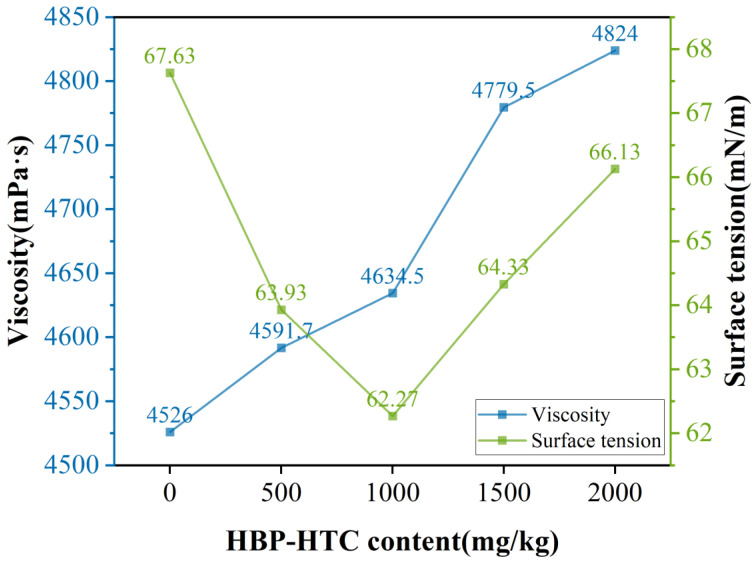
Viscosity and surface tension of spinning solution with different HBP-HTC content levels.

The increase in surface tension as depicted in Figure 8’s growth curve indicates that as the content of HBP-HTC increases, the surface tension of the spinning solution initially decreases and then continuously increases; the viscosity of the spinning solution also gradually increases with the addition of HBP-HTC. To summarize, the integration of HBP-HTC into the TPU spinning solution does not significantly affect the polymer system, thereby ensuring the spinnability of the HBP-HTC/TPU mixed spinning solution.

### 3.3. Preparation and Characterization of HBP-HTC/TPU Fiber Membranes

**Figure 9:**
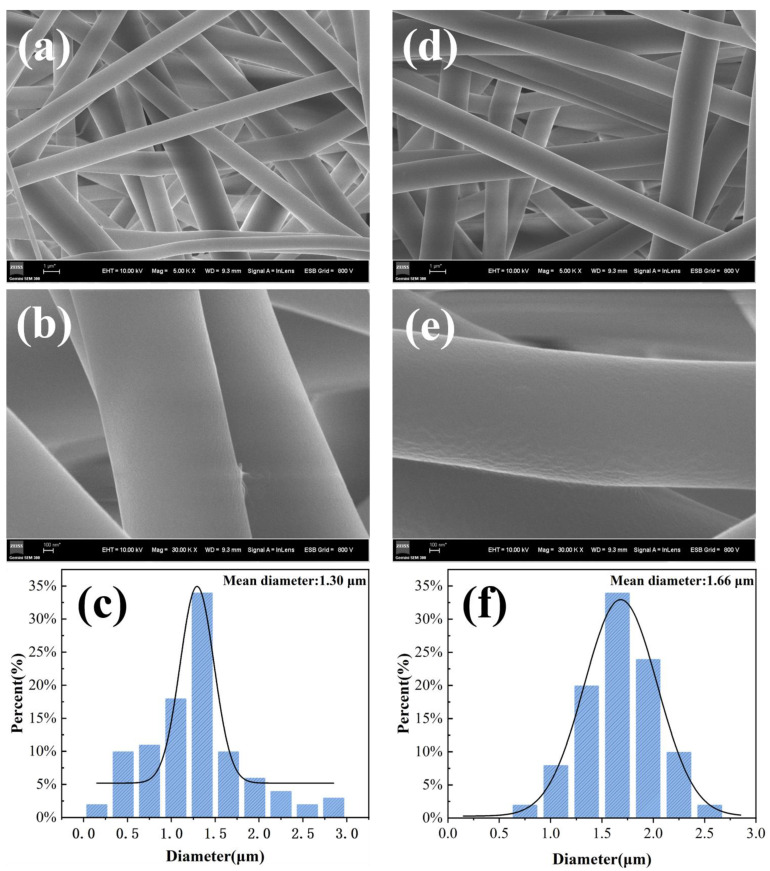
High- and low-power SEM images and diameter distribution of the fiber membrane before and after the addition of HBP-HTC, pure TPU fiber membrane: (**a**) low power, (**b**) high power, and (**c**) diameter distribution; HBP-HTC/TPU fiber membrane: (**d**) low power, (**e**) high power, and (**f**) diameter distribution.

Figure 9 illustrates that under the same electrospinning conditions, the micromorphology of the fiber membrane before and after the addition of HBP-HTC is similar, with fibers exhibiting a smooth and clean surface. This indicates that the incorporation of HBP-HTC has a minimal impact on the surface morphology of the TPU fiber membrane. If the diameter distribution is compared, the average diameter of the fibers increased from 1.30 μm to 1.66 μm after the addition of HBP-HTC. The results are consistent with the trend in viscosity changes; the addition of HBP-HTC leads to an increase in the viscosity of the spinning solution, consequently increasing fiber diameter, which is in line with the conclusions in the literature [30].

**Figure 10:**
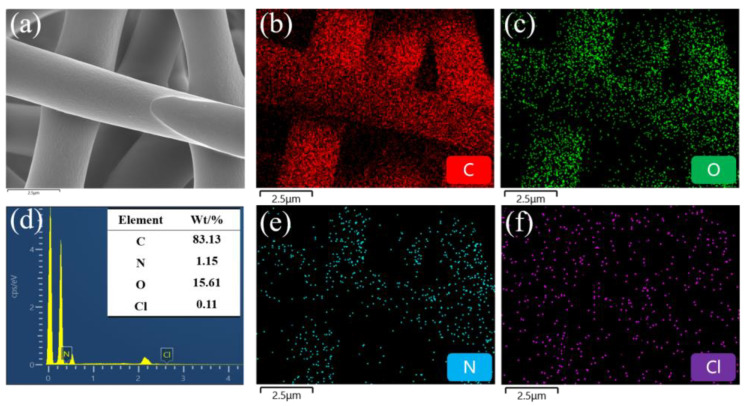
HBP-HTC/TPU fiber membranes energy spectrum: (**a**) original appearance, (**b**) C element, (**c**) O element, (**d**) element distribution, (**e**) N element, and (**f**) Cl element.

EDS is an essential tool for surface elemental analysis. Figure 10 demonstrates that the HBP-HTC/TPU fiber membrane surface contains the elements C, O, N, and Cl, with C, O, and N primarily originating from the TPU matrix, while Cl is derived from HBP-HTC. The distribution of the Cl element indicates that HBP-HTC was uniformly loaded onto the fibers.

**Figure 11:**
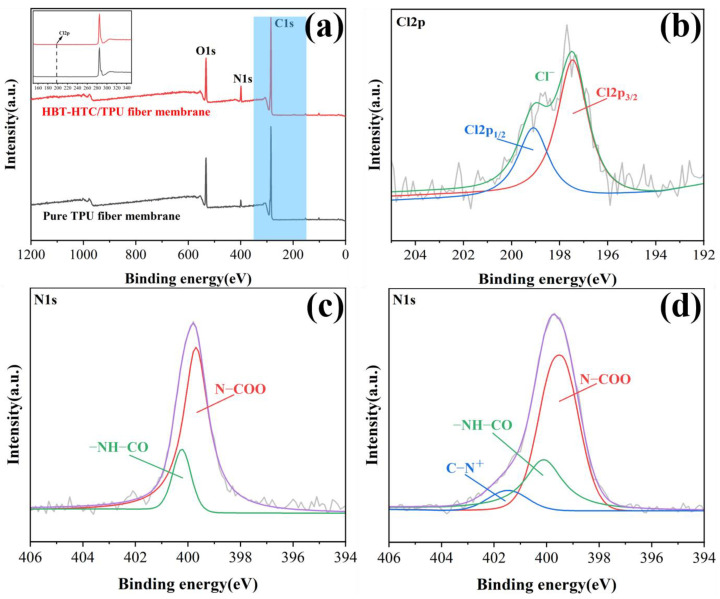
(**a**) XPS diagram of TPU fiber membranes before and after the addition of HBP-HTC, (**b**) Cl2p narrow spectrum diagram of HBP-HTC /TPU fiber membranes, (**c**) N1s narrow spectrum diagram of pure TPU fiber membranes, and (**d**) N1s narrow spectrum diagram of HBP-HTC /TPU fiber membranes.

To further verify the presence of HBP-HTC on the TPU fiber membrane, XPS analysis was conducted on the TPU fiber membranes before and after the addition of HBP-HTC. The initial survey spectrum, as illustrated in Figure 11a, revealed the presence of the characteristic peaks for C1s and O1s at 284.3 eV and 531.1 eV, specifically, in both the pure TPU fiber membrane and the HBP-HTC/TPU fiber membrane. Additionally, the HBP-HTC/TPU fiber membrane displayed a characteristic peak for Cl2p at 198.7 eV. From the synthesis process of HBP-HTC, it is known that HBP-HTC contains the Cl element. Therefore, deconvolution fitting was performed, as seen in Figure 11b. According to the literature [31], the binding energies for the free Cl^−^ characteristic peaks Cl2p_1/2_ and Cl2p_3/2_ are 197.5 eV and 199.1 eV, respectively. This confirms that the results are consistent with the EDS analysis, corroborating that HBP-HTC was successfully loaded onto the TPU fibers.

Secondly, it is evident from the N1s characteristic peak in Figure 11a that the intensity of the N1s peak for the HBP-HTC/TPU membrane is significantly higher than that for the pure TPU fiber membrane. This indicates that a substantial amount of HBP-HTC was loaded onto the fiber membrane, further increasing the nitrogen content. Further analysis through the deconvolution of the N1s peak, as illustrated in Figure 11c,d, demonstrates that the predominant energy binding at 399.5 eV and 400.1 eV is attributed to the carboxamide (N–COO) and amide (–NH–CO) groups within the TPU structure [32]. Additionally, a new nitrogen peak with a binding energy of 401.5 eV appears in Figure 11d, which is assigned to the nitrogen cation of the quaternary ammonium salt [33,34]. This confirms that the surface of the HBP-HTC/TPU fiber membrane possesses the antimicrobial quaternary ammonium salt structure.

Based on previous investigations into the electrospinning process of TPU membranes [35], it can be concluded that when the spinning voltage is within the range of 8–24 kV, the average diameter of the fibers obtained directly after electrospinning is distributed between 1.70–2.53 μm. The electrospinning process is relatively smooth, and through preliminary experiments in this study, it has been essentially determined that when the spinning voltage exceeds 18 kV, the fiber morphology is quite favorable. Therefore, by using the single-factor analysis method, with the HBP-HTC content in the spinning solution set at 1500 mg/kg and the electrospinning drum rotation speed set at 100 rpm, the effects of different spinning potentials (18, 20, 22, and 24 kV) on the structure and diameter distribution of the HBP-HTC/TPU fiber membranes were investigated.

**Figure 12:**
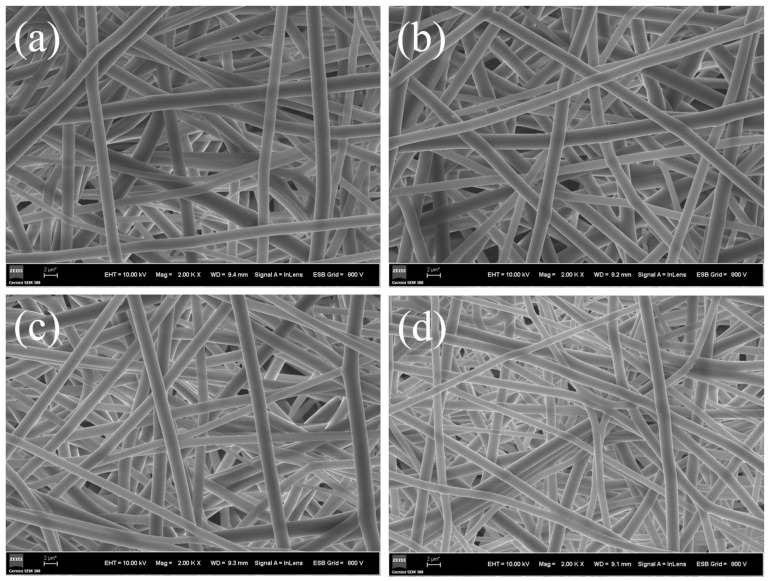
SEM of fiber membranes at different spinning voltages: (**a**) 18 kV, (**b**) 20 kV, (**c**) 22 kV, and (**d**) 24 kV.

**Figure 13:**
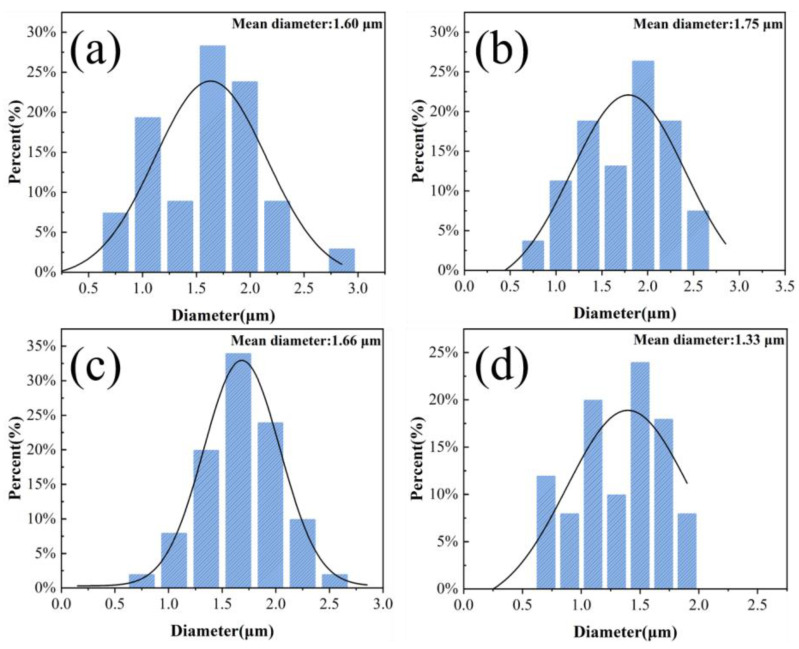
Diameter distribution of fiber membranes at different spinning voltages: (**a**) 18 kV, (**b**) 20 kV, (**c**) 22 kV, and (**d**) 24 kV.

The SEM images and diameter distribution graphs of the fiber membranes prepared under different spinning voltages are shown in Figure 12 and Figure 13. From the SEM images, it can be observed that under various spinning voltages, the fibers are well formed, exhibiting an interwoven mesh structure that ensures uniform mechanical properties in all directions. Further analysis of the diameter distribution reveals that when the spinning voltage is set to 24 kV, although the average fiber diameter decreases, the unevenness of the diameter significantly increases. This is attributed to the higher voltage enhancing the jet velocity due to the electrostatic force; however, at a fixed receiving distance, the contact time between the jet and the drum is greatly reduced, leading to an insufficient stretching of the jet and, thus, increasing the unevenness of the fiber diameter, evident as a broader span in the diameter distribution. When the spinning voltage is less than 22 kV, the average fiber diameter remains at around 1.70 μm, with the fiber membrane produced at 22 kV voltage showing a more concentrated fiber diameter distribution, averaging at 1.66 μm. Therefore, 22 kV was selected as the optimal spinning voltage.

In addition to the spinning voltage, the rotation speed of the collection drum also has a significant impact on the morphology of the fiber membrane [36]. Generally speaking, increasing the rotation speed can improve the orientation of the fibers. However, for bioprotective materials, an irregularly interwoven fiber network is the ideal distribution. Therefore, determining the rotation speed of the acceptance drum by observing the fiber distribution is crucial. The HBP-HTC content in the spinning solution was set at 1500 mg/kg, with the electrospinning voltage established at 22 kV, to investigate the effects of different drum rotation speeds (100, 500, and 1000 rpm) on the morphology and diameter distribution of the HBP-HTC/TPU fiber membranes.

**Figure 14:**
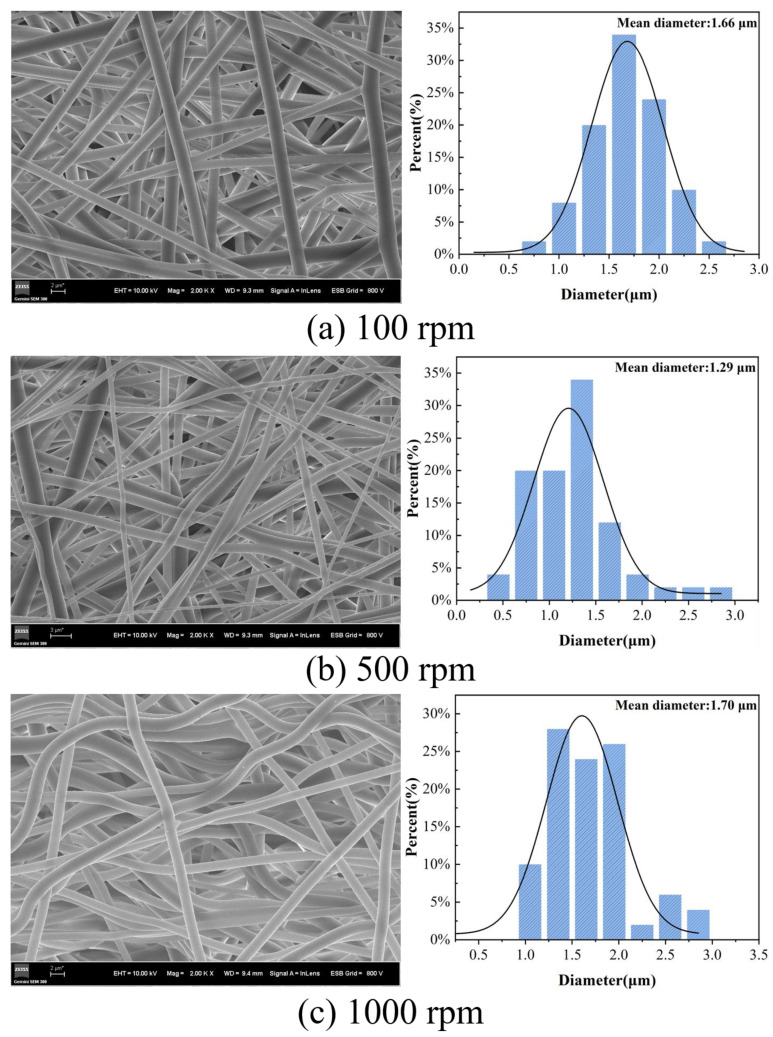
SEM and diameter distribution of fiber membranes at different drum speeds: (**a**) 100 rpm, (**b**) 500 rpm, and (**c**) 1000 rpm.

The SEM images and diameter distribution graphs of the fiber membranes prepared at different drum rotation speeds are shown in Figure 14. The diameter distribution statistics reveal that when the drum rotation speed increases from 100 to 500 rpm, the average fiber diameter decreases from 1.66 μm to 1.29 μm. It is proposed that the reduction in the fiber diameter is a consequence of the enhanced stretching of the jet at the same voltage, which is a result of the increased drum speed. However, the increased rotation speed also reduces the stability of the drum’s rotation, leading to increased fiber unevenness [37]. From the SEM image in Figure 14c, it is evident that when the speed is increased to 1000 rpm, the fibers become curved, and the overall fiber orientation is enhanced, resulting in further enlargement of the porosity. Additionally, the unevenness of the fibers is exacerbated. This occurs because adjusting the drum rotation speed under the same conditions can result in uniaxially oriented fibers; when the drum rotation speed exceeds the fiber deposition rate, curved fibers are obtained. At low rotation speeds, the fiber diameter distribution is concentrated, exhibiting an interwoven network structure; hence, 100 rpm was selected as the optimal drum rotation speed.

**Figure 15:**
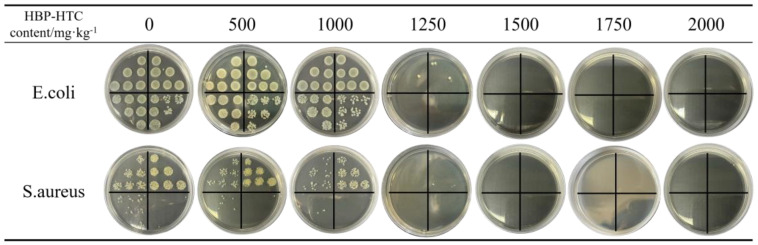
Antibacterial effect of different HBP-HTC content.

Table 1 and Figure 15 illustrate the antimicrobial performance of TPU fiber membranes against *E. coli* and *S. aureus* at different HBP-HTC content levels. The antimicrobial property of HBP-HTC/TPU fiber membranes relies primarily on the amine-terminated hyperbranched polymer quaternary ammonium salt, where the positively charged ammonium ions at the termini electrostatically adsorb onto the negatively charged bacterial cell surfaces. This interaction leads to the rupture and thinning of the cell membranes, causing the leakage of intracellular materials and, ultimately, the destruction of the osmotic gradient within the cells, resulting in cell inactivation. According to the analysis in the preceding text, HBP-HTC has been uniformly loaded onto the fiber membranes; hence, when bacteria come into contact with the fiber membranes, they are inactivated.

The data from Table 1 indicate that at an HBP-HTC content of 1000 mg/kg, the inhibition rates against *E. coli* and *S. aureus* are 42.19% and 39.76%, respectively. However, when the HBP-HTC content is ≥1500 mg/kg, the inhibition rates against both *E. coli* and *S. aureus* are ≥99.99%. According to GB/T 20944.3—2008 “Evaluation of Antimicrobial Activity of Textiles”, an inhibition rate ≥ 70% for *S. aureus* and *E. coli* confirms the antimicrobial efficacy of a sample. This suggests that the HBP-HTC/TPU fiber membrane possesses excellent antimicrobial properties, which is beneficial for its application and development in the field of medical protective materials.

Filtering performance, waterproof performance, and moisture vapor transmission tests were conducted on both pure TPU fiber membranes and HBP-HTC/TPU fiber membranes with a content of 1500 mg/kg. As observed in Table 2, the TPU fiber membranes before and after the addition of HBP-HTC meet the requirements specified in GB 19082—2009 “Technical Requirements for Medical Single-Use Protective Clothing”. The moisture vapor transmission rate of the pure TPU fiber membrane is 2891.9 g·(m^2^·d)^−1^, while the rate for the HBP-HTC/TPU fiber membrane is 2711.0 g·(m^2^·d)^−1^. There is a slight decrease in moisture vapor transmission performance, potentially due to the increased fiber diameter after the addition of HBP-HTC, which leads to smaller inter-fiber pores and, consequently, a reduced moisture vapor transmission rate. However, the impact is relatively minor. It can be concluded that the incorporation of hyperbranched polymer quaternary ammonium salts does not alter the original protective properties of the fiber membrane but also endows it with active antimicrobial capabilities, further enhancing the bioprotective performance of the fiber membrane.

## 4. Conclusions

(1) The HBP-HTC/TPU fiber membrane was fabricated via a single-step electrospinning process. When a spinning voltage of 22 kV and a drum rotation speed of 100 rpm were utilized, the resultant average fiber diameter was approximately 1.66 μm, and the fibers were characterized by an optimal diameter distribution. This revealed an interwoven mesh structure under SEM.
(2) Upon surpassing an HBP-HTC content of 1500 mg/kg within the TPU fiber membrane, the antibacterial efficacy against both *E. coli* and *S. aureus* reaches 99.99%, coupled with a filtration efficiency of 99.9%, and exhibits a hydrostatic pressure exceeding 16,758 Pa. Furthermore, the moisture vapor transmission rate reaches 2711.0 g·(m^2^·d)^−1^. Consequently, the direct incorporation of the hyperbranched polymer quaternary ammonium salt into TPU followed by electrospinning yields a fiber membrane endowed with superior protective attributes, thereby holding promising potential for application in the domain of medical protective textiles.

## Figures and Tables

**Table 1:** Antibacterial rate under different HBP-HTC content.

HBP-HTC Content/mg·kg^−1^	Inhibition Rate of *E. coli*/%	Inhibition Rate of *S. aureus*/%
0	0	0
500	13.84	12.19
1000	42.19	39.76
1250	99.98	99.94
1500	99.99	99.99
1750	99.99	99.99
2000	99.99	99.99

**Table 2:** Filter performance and waterproof and moisture permeability of TPU fiber membranes before and after adding HBP-HTC.

Sample	Filtration Efficacy/%	Water Resistance/Pa	Moisture Vapor Permeability/[g·(m^2^·d)^−1^]
Pure TPU fiber membrane	99.9	>16,758	2891.9
HBP-HTC/TPU fiber membrane	99.9	>16,758	2711.0

## Data Availability

Data are contained within the article.
